# 

**Published:** 1910-09

**Authors:** 


					CONTENTS. Page
Post-Graduate Study.
By J. M. Rattray, M.A.., M.D.
193
Cases of Leukaemia treated by X-Rays.
By J. Michell Clarke, M.A., M.D. Cantab., r.K.C.ir.
208
A Brief Account of Splenectomy with a Case of Simple Hyper-
troDhv of the Sple n treated by Operation.
iJy JAMES OWAIN,
M.S., M.D. Lond., F.R.C.b.
223
Radiographic Estimation of Simple Enlargement by means of
Visible Shadows.
By William Cotton, M.A., M.D., D.Jr.rL.
226
A Case of Anomalous Cutaneous Eruption showing a Bacillus
morphologically corresponding with the Klebs-Lceffler, and
clearing: up under Diphtheritic Antitoxin.
B y W. 1VENNETH
Wills, M.A., M.B., B.C. Cantab.
231
Progress of the Medical Sciences :
Medicine.
J. R. Charles, M.A., M.B., B.C. Cantab.
235
Surgery.
J. Lacy Firth, M.D., M.S. Lond.
23?
Dermatology.
J. A, Nixon, B.A., M.B., B.C. Cantab.
246
Reviews of Books
247
Editorial Notes
Notes on Preparations for the Sick
The Library of the Bristol Medico-Chirurgical Society   274
Obituary Notices
276
iT
Local Medical Notes

				

